# High‐Density Electroencephalography Detects Spatiotemporal Abnormalities in Brain Networks in Patients With Glioma‐Related Epilepsy

**DOI:** 10.1111/cns.70396

**Published:** 2025-04-18

**Authors:** Jiajia Liu, Jiawei Shi, Ke Li, Lei Wang, Gan You, Yinyan Wang, Xing Fan, Tao Jiang, Hui Qiao

**Affiliations:** ^1^ Department of Neurophysiology Beijing Neurosurgical Institute, Capital Medical University Beijing China; ^2^ Department of Neurosurgery Beijing Tiantan Hospital, Capital Medical University Beijing China

**Keywords:** brain network, glioma‐related epilepsy, graph theory, high‐density electroencephalography, microstate analysis

## Abstract

**Aims:**

The current study aimed to investigate brain network abnormalities in glioma‐related epilepsy (gre) patients through high‐density electroencephalography (eeg) data analysis.

**Methods:**

The study included 35 patients with newly diagnosed frontal gliomas. All participants underwent 128‐channel resting‐state EEG recordings before surgery. Afterward, graph theory and microstate analyses were performed, and the resulting metrics were compared between patients with GRE and those without GRE.

**Results:**

The network topology analysis demonstrated that the GRE group had a higher clustering coefficient, global efficiency, and local efficiency; a lower characteristic path length; and a higher small‐worldness coefficient than the non‐GRE group (adjusted *p* < 0.05 for all). Additionally, the microstate analysis indicated that the GRE group had lower occurrence and global explained variance of microstate E and higher global explained variance of microstate D (adjusted *p* < 0.05 for all). Moreover, the occurrence of microstate D was significantly negatively correlated with the maximum tumor diameter in the non‐GRE group (*r* = −0.542, *p* = 0.009).

**Conclusion:**

The current study revealed specific brain network abnormalities in GRE patients based on graph theory and microstate analyses of resting‐state high‐density EEG data. These findings can enhance our comprehension of the mechanisms behind GRE and offer potential biomarkers for improving individualized management of glioma patients.

## Graphical abstract

High‐density EEG analysis reveals that glioma‐related epilepsy (GRE) patients exhibit unique brain network dynamics, including increased local segregation and global integration, suggesting compensatory mechanisms for maintaining network stability. Reduced microstate E occurrence highlights emotional burdens, underscoring the need for psychological interventions. These findings offer insights into GRE pathophysiology and potential biomarkers for personalized management.

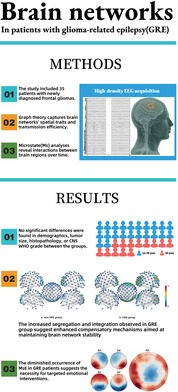

## 1 Introduction

Glioma‐related epilepsy (GRE) generally **refers** to epileptic seizures secondary to gliomas. It has been recognized as a common comorbidity of glioma, interacting with tumor progression and markedly diminishing patients' quality of life [1, 2, 3]. Recent studies have uncovered some shared mechanisms between epileptogenesis and tumor development in glioma patients, such as IDH mutation and ion channel dysfunction [4, 5]. However, the mechanisms of GRE are still not fully understood.

In recent decades, epilepsy has increasingly been considered a network‐level disorder, and extensive relevant research has been conducted based on brain imaging and electrophysiology. Accumulating evidence now indicates that tumors exert a global influence on brain networks, which may lead to tumor‐induced seizures [6, 7, 8, 9]. Accordingly, investigating the brain network characteristics in GRE patients is undeniably crucial for elucidating the mechanisms of GRE and its association with gliomas.

Functional magnetic resonance imaging (fMRI) has been employed in numerous brain network investigations due to its outstanding spatial resolution [10, 11]. Nevertheless, the basis of the technique is blood oxygenation level‐dependent responses, making it show irreparably low temporal resolution and can only indirectly measure brain activity. By contrast, electroencephalography (EEG) can directly measure neuronal activity and thus provides superior temporal resolution. On the premise of improving spatial resolution through multichannel acquisition, EEG is clearly a preferred choice for functional brain network study.

In the current study, we aimed to investigate the functional brain network mechanism of GRE by analyzing high‐density EEG data, and graph theory and microstate analysis were applied as the main analytical methods. Graph theory serves as a mathematical framework for studying graphs comprising nodes and edges (connections between nodes), and the spatial characteristics and information transmission efficiency within brain networks can be reflected by global topological and connectivity metrics [12, 13]. Microstates (Ms) are transient, stable potential distribution patterns in EEG signals, and the duration and transition frequency of these patterns may reveal interactive communications between distinct brain regions across multiple time scales [14, 15, 16]. We expected that GRE patients would show distinct characteristic modifications in graph theory and microstate metrics, which can help further understand the mechanisms of GRE and improve the personalized management of glioma patients.

## 2 Materials and Methods

**FIGURE 1:**
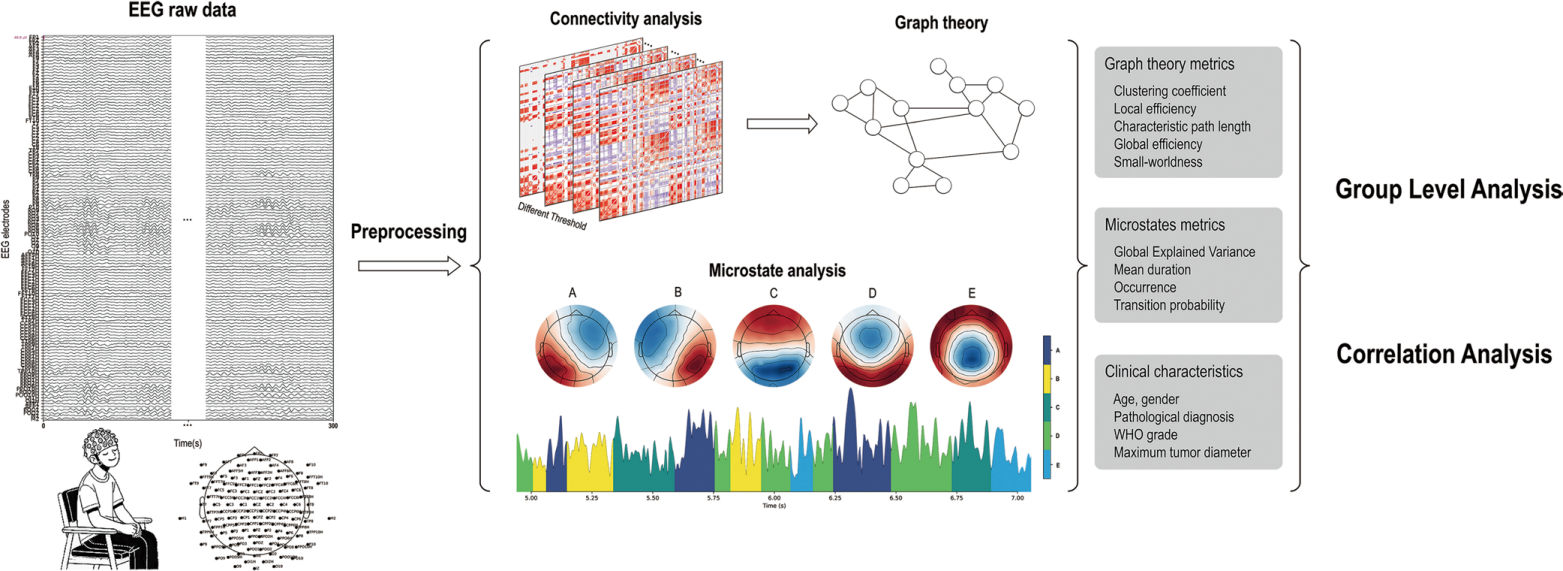
Analysis pipeline.

The analysis pipeline is summarized in Figure 1.

### 2.1 Patients

Data from frontal glioma patients treated at Beijing Tiantan Hospital between July 2023 and July 2024 were retrospectively reviewed. The inclusion criteria were as follows: (1) Aged between 18 and 60 years; (2) Right‐handed; (3) With detailed medical history and preoperative MRI data; (4) Underwent 128‐channel EEG examination following preoperative scalp preparation; (5) Histopathologically confirmed as newly diagnosed adult‐type diffuse gliomas. The exclusion criteria were as follows: (1) With multilobular tumors; (2) With neurological diseases other than gliomas and GRE; (3) Unable to comply with EEG recording standards or with poor quality of EEG recording. The demographic and clinical data, including basic information, initial symptoms, symptom duration, and lesion side, were retrieved from the hospital's Electronic Medical Record System. The maximum diameter of the tumor was assessed by preoperative MRI (within 2 weeks before surgery). The pathological diagnosis for each patient was performed according to the 2021 World Health Organization (WHO) classification of central nervous system tumors [17]. Seizure assessments were performed according to the actual application of ILAE and brain tumor‐related epilepsy assessment [18].

### 2.2 EEG Data Acquisition and Preprocessing

EEG signals were recorded by a 128‐channel EEG system (NSH0128, Neuracle Technology Changzhou Co. Ltd., Jiangsu, China). Electrode placement followed the international 10–05 system, and data were sampled at 2048 Hz. Impedances for all electrodes were maintained below 5 KΩ. Participants were instructed to relax, close their eyes, and remain seated in a quiet, dimly lit room without performing any tasks for 30 min of continuous EEG recording. An experienced neurophysiologist visually monitored the entire process and marked interference events.

Data preprocessing was performed with Python (Version 3.11.4, https://www.python.org) through the MNE‐Python package (Version 1.5.0, https://mne.tools). A digital finite impulse response (FIR) filter was applied with the bandpass between 1 and 70 Hz, while a zero‐phase notch filter was used to remove the 50 Hz hum. The down‐sampling of the EEG was set to 250 Hz. Experienced EEG experts visually identified bad channels, after which independent component analysis (ICA) was applied to remove artifacts due to eye movements, muscle activity, and cardiac interference. Two experts independently reviewed the identified artifact components; in cases of disagreement, a third expert was consulted to resolve discrepancies. Following artifact removal, bad segments were marked and excluded, and bad channels were interpolated. To ensure the EEG data can accurately reflect real brain‐source signals, we selected 5‐min continuous data segments with less than 10% bad segments for further analysis.

### 2.3 Functional Connectivity and Graph Analysis

Functional connectivity analysis was conducted using the FieldTrip toolbox (Version 20,240,309, https://www.fieldtriptoolbox.org) in MATLAB (Version R2023b). The correlations between signals from 128 sensor channels were calculated by FieldTrip's built‐in functions, generating a 128 × 128 Pearson correlation coefficient matrix. The Pearson correlation coefficient is a standard measure to identify the linear correlation between signals recorded from two electrodes.

The network graph theory metrics were calculated and processed by MATLAB using the Brain Connectivity Toolbox (https://sites.google.com/site/bctnet). We applied thresholding to the matrix at different levels to reduce the interference of fragmented information and increase the stability of calculation results [19]. Using a stricter threshold (i.e., requiring high correlation to be considered a functional link) results in fewer edges and weaker network connectivity, potentially making some nodes disconnected. Conversely, using a looser threshold (i.e., accepting low correlations as indicators of functional relationships between nodes) generates networks with denser connections but random topology [20, 21]. Ideally, the network should be evaluated across a broad range of thresholds, so our study used thresholds from 0.1 to 0.9 in intervals of 0.1. We then calculated network metrics of the correlation‐weighted symmetric matrices at different thresholds, that is, global efficiency (gE), characteristic path length (Lp), clustering coefficient (Cp), local efficiency (locE), and small‐worldness coefficient, and then conducted group‐level statistical tests. The interpretations and formulas for these critical metrics can be found in Table 1.

**TABLE 1:** Terminology and metrics for graph theory and microstate analysis.

Terminology	Definition
**Graph theory**	
Node	The vertices in the network. In this study, they are EEG electrodes
Edge	Connections between nodes. In this study, between EEG electrodes
Small‐worldness Coefficient (*σ*)	It is the normalized ratio between the clustering coefficient and average path length. When it exceeds 1, the studied network retains the small‐worldness

Global efficiency (gE)	The average inverse of the shortest path lengths between all pairs of nodes The calculation formula is given as: $\mathrm{gE}=\frac{1}{n}\sum_{i\in N}\frac{\sum_{j\in N,j\neq i}{\left(d_{\mathrm{ij}}^{w}\right)}^{-1}}{n-1}$
Characteristic path length (Lp)	The average shortest path length between all pairs of nodes in a network The calculation formula is given as follows: $\mathrm{Lp}=\frac{1}{n}\sum_{i\in N}\frac{\sum_{j\in N,j\neq i}d_{\mathrm{ij}}^{w}}{n-1}$

Clustering coefficient (Cp)	The ratio of the number of actual connections between a node's neighbor nodes in the network to the maximum number of possible connections between these neighbors The calculation formula is given as follows: $\mathrm{Cp}=\frac{1}{n}\sum_{i\in N}\frac{2t_{i}^{w}}{k_{i}\left(k_{i}-1\right)}$
Local efficiency (locE)	Same as gE, but computed on a nodal basis using information about path length between the node and its neighbors The calculation formula is given as follows: $\text{locE}=\frac{1}{n}\sum_{i\in N}\frac{\sum_{j,h\in N,j\neq i}{\left(w_{\mathrm{ij}}w_{\mathrm{ih}}{\left[d_{\mathrm{jh}}^{w}\left(N_{i}\right)\right]}^{-1}\right)}^{1/3}}{k_{i}\left(k_{i}-1\right)}$
**Microstate analysis**	
Mean duration	The average length of time that a network remains in a particular microstate before transitioning to another state
Occurrence	The frequency with which a particular microstate appears within a given period of time
Global explained variance (GEV)	The sum of the explained variances weighted by the Global Field Power at each moment in time
Transition probability	The normalized measure of how frequently transitions occur between different microstates

*Note:* *n* is the total number of nodes; $d_{\mathrm{ij}}^{w}$ is the weighted shortest path length between nodes and; $d_{\mathrm{jh}}^{w}$ is the weighted shortest path length between nodes *j* and *h* within the neighborhood subgraph induced by the neighbors of node; *t*_*i*_^*w*^ represents the number of weighted triangles involving node *i*; *k*_*i*_ is the degree of node *i*; *k*_*i*_ (*k*_*i*_ *− 1*) denotes the possible number of triangles between node *i*'s neighbors; *w*_*ij*_ and *w*_*ih*_ are the weights of the edges between node and its neighbors and ℎ, respectively.

### 2.4 EEG Microstate Analysis

The Python package Pycrostates (Version 0.5.0, https://pycrostates.readthedocs.io) in Python was applied to calculate microstate metrics. The K‐means clustering algorithm was applied to cluster scalp potential topographies at each moment of 5‐min continuous EEG recordings for each patient. By enumerating different numbers of cluster centers and using various metrics to evaluate clustering quality, we determined that setting the number of cluster centers to five was optimal. After 300 iterations of clustering, we obtained five topographic map templates, which were then compared and categorized according to previous studies, labeled as A, B, C, D, and E [16, 22]. Subsequently, we performed a back‐fitting of the original EEG data to calculate each microstate's mean duration, occurrence, global explained variance (GEV), and transition probability. To ensure the microstate sequences were more stable and continuous, we used a smoothing algorithm during back‐fitting, with a smoothing factor set to 10 and a smoothing window size of four. Finally, we obtained various metrics for each microstate for each patient and conducted group‐level statistical comparisons.

### 2.5 Statistical Analysis

The R software was used for statistical tests and plotting (Version 4.2.3, www.r‐project.org). The Shapiro–Wilk test for normality was initially performed to determine whether the clinical‐pathological features, network properties, and microstate metrics in both groups followed a normal distribution. Data that met the normality assumption were compared using an independent samples t‐test. For data that did not meet the normality assumption, the nonparametric Wilcoxon rank‐sum test was applied. The differences in categorical variables between groups were compared using the chi‐square test. Subsequently, Pearson correlation analyses were conducted to explore the relationships between brain network properties, microstate metrics, and clinical indicators. Linear regressions were plotted for correlations that reached statistical significance. All statistical results were adjusted for multiple comparisons using the false discovery rate (FDR) correction. The significance level (α) was set at 0.05, and all reported P‐values were obtained after applying the multiple comparison correction.

## 3 Results

### 3.1 Participant Demographics

A total of 35 patients were finally included, and 13 of them were diagnosed with GRE. The clinical and pathological features of the GRE and non‐GRE groups are summarized in Table 2. There were no significant differences in age, gender, maximum tumor diameter, histopathology, and WHO CNS grade between the two groups (both *p* > 0.05).

**TABLE 2:** Clinical and pathological features in the patient cohort.

Clinical and pathological features	GRE (*n* = 13)	Non‐GRE (*n* = 22)	*p*
Gender			0.31^b^
Male	8	9	
Female	5	13	
Age, years^a^	36.69 ± 9.23	40.82 ± 11.23	0.27^b^
Maximal diameter, mm^a^	38.23 ± 11.55	39.77 ± 12.81	0.72^a^
Pathological diagnosis			0.90^d^
Astrocytoma	7	9	
Oligodendroglioma	4	9	
Glioblastoma	2	4	
WHO CNS grade			1.00^b^
Low grade	7	12	
High grade	6	10	

Abbreviations: GRE, the group of patients with glioma‐related epilepsy; non‐GRE, the group of patients without glioma‐related epilepsy.

^a^ Values are means ± SD.

^b^ Determined with the chi‐square test.

^c^ Determined with the T‐test.

^d^ Determined by Fisher's exact test.

### 3.2 Graph Theory Metrics

In this study, five common indicators were mainly analyzed, namely Cp, Lp, gE, locE, and small‐worldness coefficient. Cp and locE assess the local structure and function of the brain network. Higher values indicate better connectivity, information flow efficiency, and tighter subnetworks. Lp and gE evaluate the integration of the brain network. Higher gE and shorter Lp reflect greater integration and improved information transmission efficiency. The small‐worldness coefficient is a measure of the balance of segregation and integration in a network.

**FIGURE 2:**
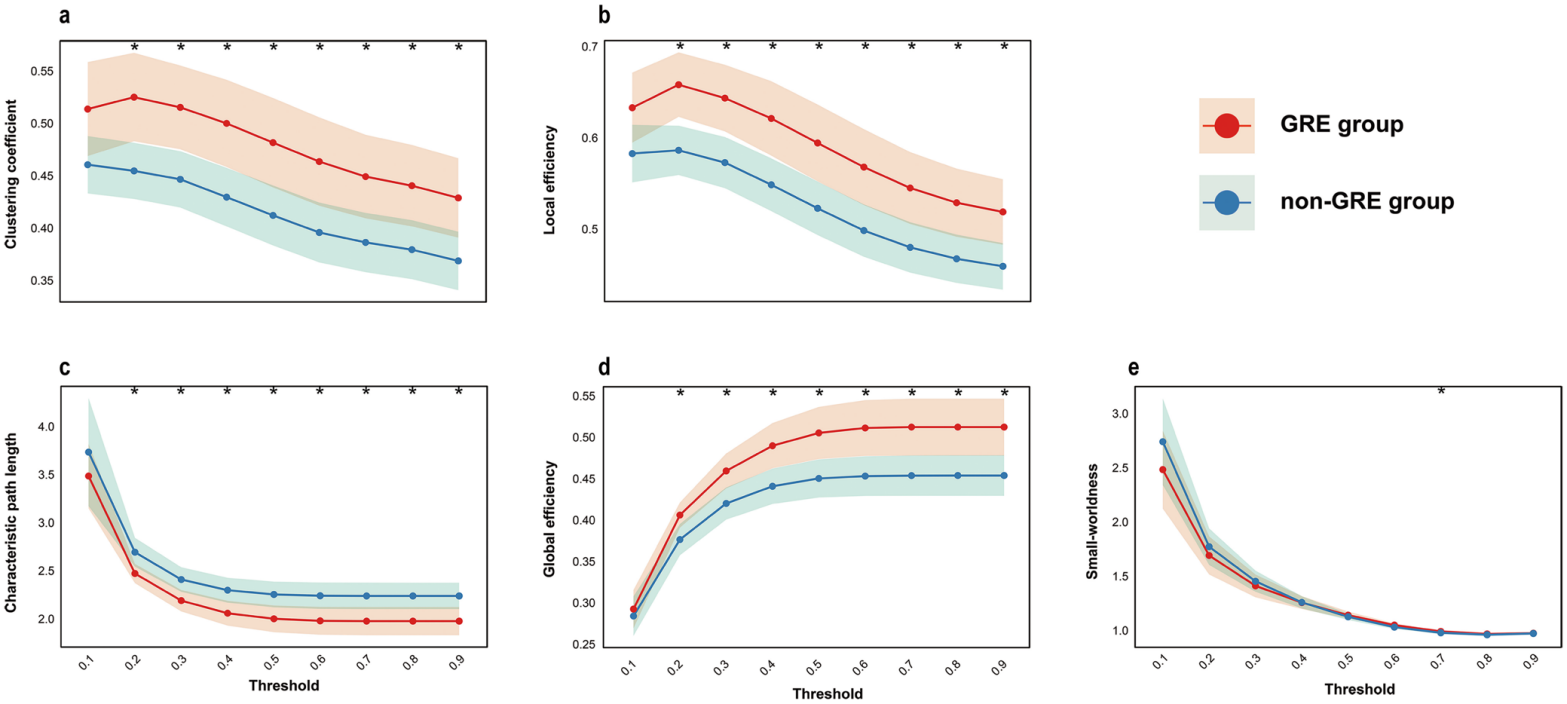
Comparison of graph‐based functional connectivity metrics at different thresholds between the two groups. Panels a, b, c, d, and e show comparisons of the clustering coefficient, local efficiency, characteristic path length, global efficiency, and small‐worldness coefficient between the two groups, respectively. Compared with the non‐GRE group, the GRE group has a significantly higher clustering coefficient, global efficiency and local efficiency, and shorter path length (both > 0.2 scale threshold). In addition, the small‐worldness coefficient only showed differences at a threshold of 0.7. GRE, the group of patients with glioma‐related epilepsy; non‐GRE, the group of patients without glioma‐related epilepsy. *p < 0.05 after false discovery rate correction.

The whole‐brain functional connectivity maps based on sensor‐level EEG correlation of the GRE and non‐GRE groups are shown in Supplementary Figure S1. The graph theory metrics were compared between the GRE and non‐GRE groups at various thresholds (Figure 2). Compared with the non‐GRE group, the metrics Cp, gE, and locE were significantly higher (*p* < 0.05) in the GRE group under the vast majority of thresholds (from 0.2 to 0.9). In contrast, Lp was significantly lower (*p* < 0.05) in the GRE group under the same condition. Notably, the small‐worldness coefficient Sigma exhibited significant differences between the groups only at a threshold of 0.7, with the value in the GRE group being slightly higher than that in the non‐GRE group (*p* < 0.05, both Sigma < 1). The overall trends of all metrics gradually decreased with increasing thresholds except for gE, which was the opposite. The detailed statistical test results are provided in Supplementary Table S1.

Overall, the results showed that compared with the non‐GRE group, the brain network of GRE showed characteristics of high integration and separation. Additionally, although both the GRE and non‐GRE groups had lost small‐world properties, the GRE group showed network characteristics that were closer to these properties. These findings may reflect the adaptive responses of brain networks to glioma‐related epilepsy.

### 3.3 EEG Microstate Analysis

**FIGURE 3:**
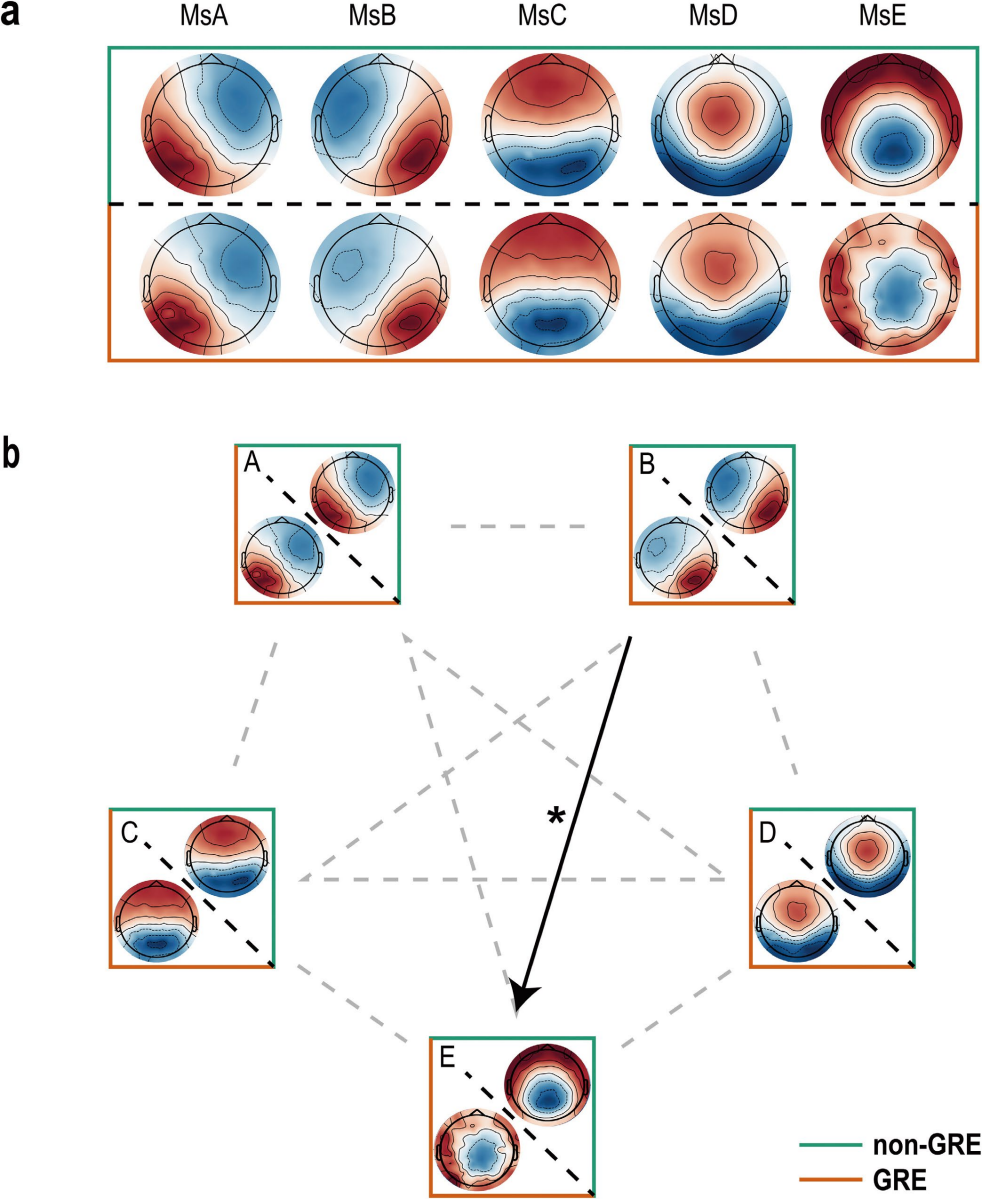
Comparison of the five microstate patterns identified by k‐means cluster analysis and the transition probabilities between these patterns in the non‐GRE and GRE groups. GRE, the group of patients with glioma‐related epilepsy; non‐GRE, the group of patients without glioma‐related epilepsy; Ms., microstate. *p < 0.05 after false discovery rate correction.

As shown in Figure 3a, by enumerating different numbers of cluster centers and using various metrics to evaluate the clustering quality, we determined that setting the number of cluster centers to five was optimal. The evaluation metrics for the cluster centers are detailed in Supplementary Figure S2. The five resulting topographic patterns were compared and classified with previous studies [16, 22]. The topography with a right frontal to left posterior configuration was labeled as MsA; the topography with a left frontal to right posterior configuration was labeled as MsB; the topography with a symmetrical anterior–posterior configuration was labeled as MsC; the topography with a frontal, central configuration was labeled as MsD; and the topography with a centro‐parietal maximum was labeled as MsE.

**FIGURE 4:**
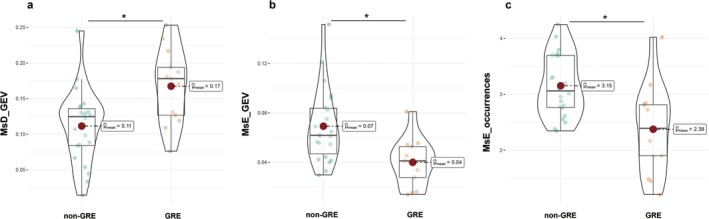
Comparison of the temporal dynamics of the five microstate patterns between the non‐GRE and GRE groups. Panels a, b, and c are comparisons of GEV for MsD, GEV for MsE, and occurrences for MsE between the two groups, respectively. GRE, the group of patients with glioma‐related epilepsy; non‐GRE, the group of patients without glioma‐related epilepsy; MsD, microstate D; MsE, microstate E; GEV, Global Explained Variance. *p < 0.05 after false discovery rate correction.

The metrics of the two groups' microstate analysis and the direct transition probabilities between each microstate pattern are shown in Figures 3b and 4. The GEV of MsD in the GRE group was significantly higher than in the non‐GRE group(*p* = 0.029). Conversely, the GEV and occurrence of MsE in the GRE group were significantly lower than those in the non‐GRE group (*p* = 0.019 and 0.029, respectively). We also calculated the direct transition probabilities between each microstate pattern and found that the transition probability from MsB to MsE in the GRE group was significantly lower than in the non‐GRE group (*p* = 0.046). The concrete statistical test results are provided in Supplementary Tables S2 and S3.

**FIGURE 5:**
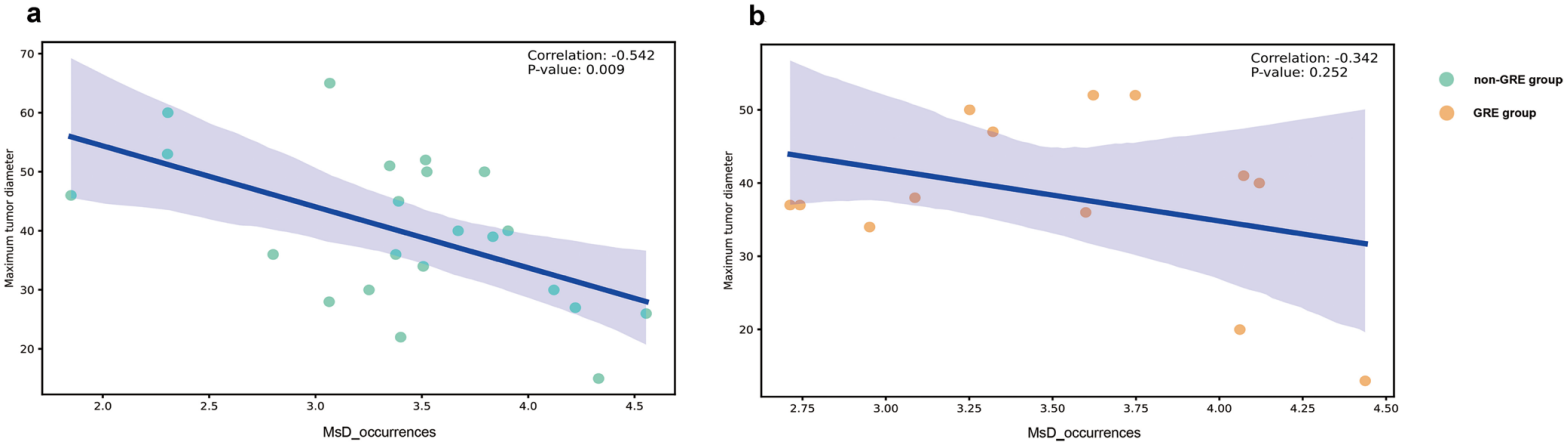
Spearman's correlation between maximal tumor diameter and the occurrence of MsD in each group. a. the group of patients with glioma‐related epilepsy; b. the group of patients without glioma‐related epilepsy. MsD, microstates D.

In addition, we found that the occurrence of MsD was significantly negatively correlated with the maximum tumor diameter in the non‐GRE group (*r* = −0.542, *p* = 0.009), while there was no such correlation in the GRE group (*p* = 0.252). The results are shown in Figure 5.

## 4 Discussion

Further understanding of brain network abnormalities in GRE patients can bring new insights into GRE mechanisms, allowing for more precise evaluation and therapy. In the current study, we compared the brain network characteristics between patients with GRE and those without and found that GRE patients showed higher gE, Cp, locE, and shorter Lp. Moreover, the occurrence and GEV of MsE were lower in the brain network activities of the GRE patients, while MsD exhibited a higher GEV.

The brain is a highly nonlinear complex network system, and local segregation and global integration are the two essential foundations of neurofunction [23]. Local segregation refers to the independent processing in specialized network subsystems and can be characterized by Cp and locE. Global integration refers to the cooperation between different network subsystems and can be characterized by gE and Lp. Furthermore, the resting brain networks are, on average, close to a segregation‐integration balance. Neurological disorders are often associated with changes in the two foundations and their balance. For instance, it has been shown that the brain networks in idiopathic epilepsy patients are typified by reduced integration and increased segregation [24, 25, 26, 27]. Here, we identified that compared with non‐GRE patients, GRE patients exhibited high segregation (higher Cp and locE) and integration (higher gE and lower Lp).

Due to the lack of healthy controls, it seems difficult to determine which group of patients has “healthier” brain networks. However, according to our previous research and clinical practice, GRE patients generally suffer from gliomas with low malignancy and thus enjoy a better survival prognosis. By contrast, non‐GRE patients often suffer from highly invasive tumors, which may result in severe destruction of brain networks, thereby disrupting the basis for the origination and propagation of epileptic activities [28, 29]. Moreover, previous studies also found that GRE patients had relatively intact structural networks while non‐GRE patients had damaged networks [30, 31]. Considering that structural networks are the physical substrate of functional networks, the functional networks of GRE patients are more likely to be in a “healthier” state. Additionally, some studies have found that epilepsy may increase the connectivity and topological properties of white matter [32, 33, 34, 35]. Our results showed GRE can increase the topological properties of functional networks, indirectly supporting the consistency of structural and functional networks.

The difference in functional networks between GRE and non‐GRE patients can be explained by the differences in neuroplasticity guided by the particular tumor growth pattern. Neuroplasticity refers to the ability of brain networks to compensate for the consequences of a lesion or injury, and the theory that glioma can lead to structural and topological plasticity has been widely accepted [36, 37]. Like stroke or brain trauma, rapidly evolving tumors typically result in limited neuroplasticity. By contrast, slow‐growing tumors generally show greater plastic potential. Notably, the seemingly “healthier” brain networks observed in GRE patients are temporary and do not imply the surgery can be postponed. Since it is a form of compensation, it will inevitably develop into decompensation without etiological treatment.

Based on our results, we could gain a new understanding of the etiology of GRE from the perspective of functional networks. Compared with non‐GRE patients, GRE patients had a “healthier” state of functional networks. Such a “healthier” state might not necessarily correlate with specific graph theory metrics but refer to a better segregation‐integration balance. We suggested that the network basis of epilepsy (maybe increased local segregation) was a consequence of functional network compensation for gliomas. In other words, the network compensation for slow‐growing gliomas could increase the epilepsy susceptibility of the local brain. It is certainly an overinterpretation, but we raise this viewpoint here and hope to have more relevant investigations in the future.

The results of microstate analysis show that the intensity (i.e., potential difference) of MsD in the GRE group at each occurrence was higher than that in the non‐GRE group, which led to a higher GEV value. GEV measures the relative contribution and explanatory power of a specific microstate in interpreting EEG signals. It is related to the intensity and explanatory power of the microstate at a time point. The result above can be interpreted as the brain activity caused by epileptic seizures becoming abnormally strong or synchronous at certain moments, which may be the high‐intensity activity exhibited by the brain in the process of continuous reorganization and adaptation in response to the damage caused by seizures. This enhancement phenomenon also exists in the language and visual networks of patients with temporal GRE [38]. In addition, in the non‐GRE group, the occurrence of MsD was negatively correlated with the maximum diameter of the tumor. It suggested that as the tumor grows, the impairment of some functional networks related to MsD (such as cognitive control [16]) becomes more apparent. This phenomenon does not exist in the GRE group. A possible explanation is that the brain networks of GRE patients show a particular ability in adaptation and reorganization, which may make them more resilient and adaptive in some aspects than the non‐GRE group.

Another significant finding is that the occurrence of MsE is lower in the GRE group. Custo et al. identified cortical areas significantly associated with MsE, including the dorsal anterior cingulate cortex, superior frontal gyrus, middle frontal gyrus, and insula [22]. These areas overlap with the source of MsC and are involved in interoception and emotional processing, symbolizing relaxation and comfort [16]. A diffusion‐weighted imaging study on frontal GRE found that the degree centrality and betweenness centrality of nodes in the middle frontal gyrus were reduced in both GRE and non‐GRE groups compared to healthy controls [39]. The middle frontal gyrus is crucial for emotional regulation, and anomalies in its node centrality may lead to abnormal amygdala activation, causing excessive anxiety in glioma patients [40, 41]. After seizure occurrence, patients often remain anxious about potential seizures and have difficulty achieving a state of relaxation. The result prompted the conclusion that GRE patients generally bear a more significant emotional burden and require more targeted emotional interventions.

This study has several limitations. First, the absence of healthy controls affected the interpretation of the results. Second, the limited sample size restricts the ability to conduct further stratified analysis and diminishes the statistical power of the existing analysis. However, despite these limitations, the positive results remain relatively reliable under strict statistical correction. To further validate and expand our findings, it is essential to integrate high‐density EEG with additional modalities, such as radiomics and clinical data, through large‐scale, prospective studies. Additionally, the presence of brain tumors poses challenges for the application of traditional high‐density EEG analysis methods, such as brain source reconstruction. Addressing these challenges is an important direction for future research.

## 5 Conclusions

In summary, graph theory and microstate analyses based on resting‐state high‐density EEG data elucidate the differences in the spatiotemporal characteristics of brain networks between GRE and non‐GRE patients. Compared with the non‐GRE group, the brain networks of the GRE group exhibit pronounced segregation and integration. Furthermore, the diminished occurrence of MsE in GRE patients suggests the necessity for targeted emotional interventions.

## Author Contributions

Conception and design of the study: H.Q., T.J., X.F.; data acquisition: J.L., J.S., K.L.; statistical analysis: J.S.; data interpretation: L.W., G.Y.; manuscript preparation: J.L., J.S.; manuscript revisions: H.Q., T.J., X.F.; funding acquisition: X.F., Y.W. All authors have reviewed the manuscript and approved the manuscript for publication.

## Ethics Statement

The study was approved by the Ethics Committee of Beijing Tiantan Hospital (KY2022‐217‐03). Written informed consent was obtained from all patients. All procedures were conducted in accordance with the ethical standards of the 1964 Declaration of Helsinki and its subsequent amendments.

## Conflicts of Interest

The authors declare no conflicts of interest.

## Supporting information


**Table S1.**Comparison of graph‐based functional connectivity indices at different thresholds between the two groups^†^.
**Table S2.**Comparison of the temporal dynamics of the five microstate patterns between the GRE and non‐GRE groups^†^.
**Table S3.**Comparison of transition probabilities between the five microstate patterns in the GRE and non‐GRE groups^†^.
**Figure S1.**Whole‐brain functional connectivity based on sensor‐level EEG correlation in each group. Group‐level connections are averaged and plotted as an edge between different channels anatomically parcellated using the Montreal Neurological Institute standard brain. For better visualization, the threshold of connections was set to edge weight = 0.8 (i.e., 80% of low‐correlation connections were removed). Each channel is indicated as a sphere. As shown in the figure, compared with the non‐GRE group, the number of connections and correlations between channels in the frontal lobe and occipital lobe of the GRE group were significantly increased. GRE, the group of patients with glioma‐related epilepsy; non‐GRE, the group of patients without glioma‐related epilepsy.
**Figure S2.**The evaluation metrics for the cluster centers. The higher the Silhouette score, Calinski‐Harabasz score, and Dunn score, the better, while the lower the Davies–Bouldin score, the better [1–4]. Taken together, clustering into five microstate patterns is most suitable.

## Data Availability

The data that support the findings of this study are available on request from the corresponding author. The data are not publicly available due to privacy or ethical restrictions.
