# Supplementary material for: High‐Density Electroencephalography Detects Spatiotemporal Abnormalities in Brain Networks in Patients With Glioma‐Related Epilepsy
Source: CNS Neurosci Ther. 2025 Apr 18;31(4):e70396. doi: 10.1111/cns.70396 (PMC12007183; doi:10.1111/cns.70396)
Supplement: Supplementary file 1 — Table S1.Comparison of graph‐based functional connectivity indices at different thresholds between the two groups†. Table S2.Comparison of the temporal dynamics of the five microstate patterns between the GRE and non‐GRE groups†. Table S3.Comparison of transition probabilities between the five microstate patterns in the GRE and non‐GRE groups†. Figure S1.Whole‐brain functional connectivity based on sensor‐level EEG correlation in each group. Group‐level connections are averaged and plotted as an edge between different channels anatomically parcellated using the Montreal Neurological Institute standard brain. For better visualization, the threshold of connections was set to edge weight = 0.8 (i.e., 80% of low‐correlation connections were removed). Each channel is indicated as a sphere. As shown in the figure, compared with the non‐GRE group, the number of connections and correlations between channels in the frontal lobe and occipital lobe of the GRE group were significantly increased. GRE, the group of patients with glioma‐related epilepsy; non‐GRE, the group of patients without glioma‐related epilepsy. Figure S2.The evaluation metrics for the cluster centers. The higher the Silhouette score, Calinski‐Harabasz score, and Dunn score, the better, while the lower the Davies–Bouldin score, the better [1–4]. Taken together, clustering into five microstate patterns is most suitable. [file CNS-31-e70396-s001.docx]

**SUPPLEMENTARY MATERIALS**

1. **Supplementary Tables**

**Supplementary Table 1. Comparison of graph-based functional connectivity indices at different thresholds between the two groups^†^**

| **Metrics** | **Threshold** | **GRE** | **Non-GRE** | **Adjusted *P*-Value** |
| --- | --- | --- | --- | --- |
| Clustering Coefficient | 0.1 | 0.51±0.07 | 0.46±0.06 | 0.05 |
|  | 0.2 | 0.52±0.07 | 0.45±0.06 | 0.02 |
|  | 0.3 | 0.52±0.07 | 0.45±0.06 | 0.02 |
|  | 0.4 | 0.50 (0.46, 0.55) | 0.44 (0.41, 0.46) | 0.01 |
|  | 0.5 | 0.49 (0.44, 0.53) | 0.42 (0.39, 0.44) | 0.01 |
|  | 0.6 | 0.47 (0.43, 0.51) | 0.40 (0.37, 0.42) | 0.01 |
|  | 0.7 | 0.45 (0.41, 0.49) | 0.39 (0.36, 0.42) | 0.01 |
|  | 0.8 | 0.44 (0.41, 0.48) | 0.38 (0.36, 0.41) | 0.01 |
|  | 0.9 | 0.43 (0.39, 0.46) | 0.37 (0.35, 0.40) | 0.01 |
| Local Efficiency | 0.1 | 0.63±0.06 | 0.58±0.07 | 0.05 |
|  | 0.2 | 0.66±0.06 | 0.59±0.06 | 0.02 |
|  | 0.3 | 0.64±0.06 | 0.57±0.06 | 0.02 |
|  | 0.4 | 0.62±0.07 | 0.55±0.07 | 0.02 |
|  | 0.5 | 0.59±0.07 | 0.52±0.07 | 0.02 |
|  | 0.6 | 0.57±0.07 | 0.50±0.06 | 0.02 |
|  | 0.7 | 0.54±0.06 | 0.48±0.06 | 0.02 |
|  | 0.8 | 0.53±0.06 | 0.47±0.06 | 0.02 |
|  | 0.9 | 0.52±0.06 | 0.46±0.06 | 0.02 |
| Global Efficiency | 0.1 | 0.29 (0.27, 0.32) | 0.29 (0.27, 0.32) | 0.95 |
|  | 0.2 | 0.41±0.02 | 0.38±0.04 | 0.02 |
|  | 0.3 | 0.46±0.03 | 0.42±0.04 | 0.02 |
|  | 0.4 | 0.49±0.05 | 0.44±0.05 | 0.02 |
|  | 0.5 | 0.51±0.05 | 0.45±0.05 | 0.02 |
|  | 0.6 | 0.51±0.06 | 0.45±0.05 | 0.02 |
|  | 0.7 | 0.51±0.06 | 0.45±0.06 | 0.02 |
|  | 0.8 | 0.51±0.06 | 0.45±0.06 | 0.02 |
|  | 0.9 | 0.51±0.06 | 0.45±0.06 | 0.02 |
| Characteristic Path Length | 0.1 | 3.40 (3.16, 3.69) | 3.40 (3.09, 3.66) | 0.95 |
|  | 0.2 | 2.43 (2.35, 2.53) | 2.62 (2.44, 2.87) | 0.04 |
|  | 0.3 | 2.13 (2.10, 2.25) | 2.33 (2.22, 2.54) | 0.01 |
|  | 0.4 | 1.98 (1.95, 2.13) | 2.22 (2.11, 2.41) | 0.01 |
|  | 0.5 | 1.93 (1.87, 2.08) | 2.16 (2.06, 2.37) | 0.01 |
|  | 0.6 | 1.91 (1.84, 2.07) | 2.15 (2.05, 2.36) | 0.01 |
|  | 0.7 | 1.91 (1.84, 2.07) | 2.15 (2.05, 2.36) | 0.01 |
|  | 0.8 | 1.91 (1.84, 2.07) | 2.15 (2.05, 2.36) | 0.01 |
|  | 0.9 | 1.91 (1.84, 2.07) | 2.15 (2.05, 2.36) | 0.01 |
| Small-worldness Coefficient | 0.1 | 2.48±0.59 | 2.74±0.90 | 0.40 |
|  | 0.2 | 1.69±0.29 | 1.77±0.38 | 0.53 |
|  | 0.3 | 1.41±0.17 | 1.45±0.22 | 0.57 |
|  | 0.4 | 1.26±0.10 | 1.26±0.13 | 0.96 |
|  | 0.5 | 1.14 (1.12, 1.15) | 1.13 (1.09, 1.17) | 0.74 |
|  | 0.6 | 1.05 (1.04, 1.05) | 1.04 (1.01, 1.05) | 0.10 |
|  | 0.7 | 0.99 (0.99, 1.00) | 0.98 (0.97, 0.99) | 0.01 |
|  | 0.8 | 0.97 (0.96, 0.97) | 0.962 (0.96, 0.97) | 0.06 |
|  | 0.9 | 0.98 (0.97, 0.98) | 0.974 (0.97, 0.98) | 0.69 |

GRE, the group of patients with glioma-related epilepsy; non-GRE, the group of patients without glioma-related epilepsy. **^†^**These data are presented as mean ± SD for normally distributed samples and as median (interquartile range, IQR) for non-normally distributed samples.

**Supplementary Table 2. Comparison of the temporal dynamics of the five microstate patterns between the GRE and non-GRE groups^†^**

| **Metrics** | **GRE** | **non-GRE** | **Adjusted *P*-Value** |
| --- | --- | --- | --- |
| Ms A |  |  |  |
| GEV | 0.07 (0.04, 0.08) | 0.06 (0.03, 0.10) | 0.96 |
| mean duration | 0.06 (0.06, 0.06) | 0.06 (0.05, 0.06) | 0.96 |
| occurrences | 2.61 ±1.07 | 2.82±0.78 | 0.93 |
| Ms B |  |  |  |
| GEV | 0.08±0.05 | 0.07±0.05 | 0.93 |
| mean duration | 0.06±0.01 | 0.06±0.01 | 0.93 |
| occurrences | 2.70±1.01 | 2.80±0.95 | 0.93 |
| Ms C |  |  |  |
| GEV | 0.18 (0.12, 0.25) | 0.14 (0.11, 0.23) | 0.96 |
| mean duration | 0.06 (0.06, 0.08) | 0.06 (0.06, 0.08) | 0.96 |
| occurrences | 3.52±0.94 | 3.64±0.77 | 0.93 |
| Ms D |  |  |  |
| GEV | 0.17±0.05 | 0.11±0.05 | 0.03 |
| mean duration | 0.07 (0.06, 0.08) | 0.06 (0.06, 0.07) | 0.12 |
| occurrences | 3.52±0.56 | 3.42±0.67 | 0.93 |
| Ms E |  |  |  |
| GEV | 0.04±0.02 | 0.07±0.03 | 0.02 |
| mean duration | 0.09 (0.05, 0.06) | 0.06 (0.06, 0.07) | 0.32 |
| occurrences | 2.38±0.79 | 3.15±0.56 | 0.03 |

GRE, the group of patients with glioma-related epilepsy; non-GRE, the group of patients without glioma-related epilepsy; Ms, microstate; GEV, global explained variance. **^†^**These data are presented as mean ± SD for normally distributed samples and as median (interquartile range, IQR) for non-normally distributed samples.

**Supplementary Table 3. Comparison of transition probabilities between the five microstate patterns in the GRE and non-GRE groups^†^**

| **Metrics** | **GRE** | **non-GRE** | **Adjusted *P*-Value** |
| --- | --- | --- | --- |
| Ms A |  |  |  |
| to MsB | 0.23±0.09 | 0.23±0.09 | 0.98 |
| to MsC | 0.30±0.08 | 0.28±0.08 | 0.98 |
| to MsD | 0.28±0.07 | 0.26±0.06 | 0.76 |
| to MsE | 0.20±0.07 | 0.24±0.05 | 0.35 |
| Ms B |  |  |  |
| to MsA | 0.22±0.09 | 0.23±0.07 | 0.98 |
| to MsC | 0.28±0.08 | 0.29±0.08 | 0.98 |
| to MsD | 0.30 (0.23, 0.36) | 0.25 (0.23, 0.27) | 0.52 |
| to MsE | 0.17 (0.13, 0.18) | 0.23 (0.20, 0.25) | 0.05^a^ |
| Ms C |  |  |  |
| to MsA | 0.21±0.10 | 0.21±0.07 | 0.98 |
| to MsB | 0.23±0.09 | 0.22±0.08 | 0.98 |
| to MsD | 0.36±0.14 | 0.30±0.09 | 0.65 |
| to MsE | 0.20±0.08 | 0.26±0.07 | 0.28 |
| Ms D |  |  |  |
| to MsA | 0.21±0.09 | 0.21±0.07 | 0.98 |
| to MsB | 0.24±0.11 | 0.21±0.08 | 0.91 |
| to MsC | 0.35±0.14 | 0.32±0.12 | 0.98 |
| to MsE | 0.19 (0.16, 0.22) | 0.24 (0.20, 0.29) | 0.15 |
| Ms E |  |  |  |
| to MsA | 0.21±0.09 | 0.21±0.06 | 0.98 |
| to MsB | 0.21±0.08 | 0.21±0.08 | 0.98 |
| to MsC | 0.30±0.11 | 0.31±0.10 | 0.98 |
| to MsD | 0.27 (0.25, 0.29) | 0.26 (0.23, 0.29) | 0.95 |

GRE, the group of patients with glioma-related epilepsy; non-GRE, the group of patients without glioma-related epilepsy; Ms, microstate. **^†^**These data are presented as mean ± SD for normally distributed samples and as median (interquartile range, IQR) for non-normally distributed samples. ^a^=0.046

1. **Supplementary Figures legends**

**
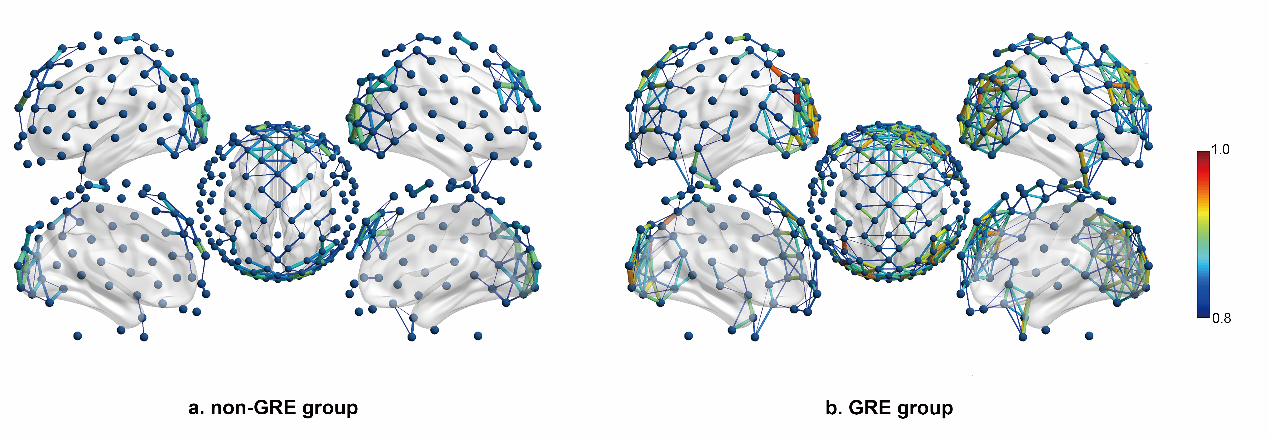
**

**Supplementary Figure S1: Whole-brain functional connectivity based on sensor-level EEG correlation in each group. Group-level connections are averaged and plotted as an edge between different channels anatomically parcellated using the Montreal Neurological Institute standard brain. For better visualization, the threshold of connections was set to edge weight = 0.8 (i.e., 80% of low-correlation connections were removed). Each channel is indicated as a sphere. As shown in the figure, compared with the non-GRE group, the number of connections and correlations between channels in the frontal lobe and occipital lobe of the GRE group were significantly increased. GRE, the group of patients with glioma-related epilepsy; non-GRE, the group of patients without glioma-related epilepsy.**


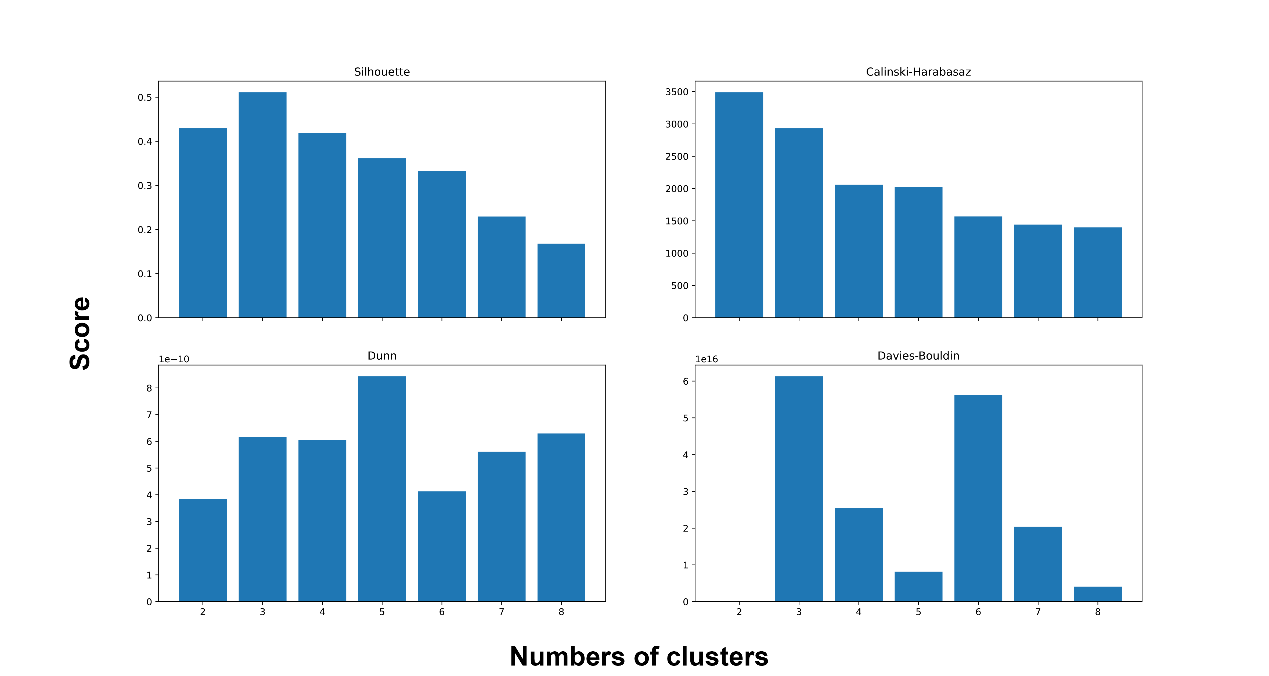


**Supplementary Figure S2: The evaluation metrics for the cluster centers. The higher the Silhouette score, Calinski-Harabasz score, and Dunn score, the better, while the lower the Davies-Bouldin score, the better.^1-4^ Taken together, clustering into 5 microstate patterns is most suitable.**

**References**

1. Rousseeuw PJ. Silhouettes: A graphical aid to the interpretation and validation of cluster analysis. *J Comput Appl Math*. 1987;20:53-65.

2. Davies DL, Bouldin DW. A Cluster Separation Measure. *IEEE Trans Pattern Anal Mach Intell*. 1979;PAMI-1(2):224-227.

3. Dunn† JC. Well-Separated Clusters and Optimal Fuzzy Partitions. *Cybern Syst*. 1974;4:95-104.

4. Caliński T, Harabasz J. A dendrite method for cluster analysis. *Commun Stat Theory Methods*. 1974;3:1-27.
